# Case report: Successful outcome of a young patient with rhabdomyolysis and shock caused by diquat poisoning

**DOI:** 10.3389/fmed.2023.1116912

**Published:** 2023-02-03

**Authors:** Yunchao Chen, Zejin Ou, Ruichang Zhang, Zhenhong Long, Rushen Fu, Shihao Tang, Zhi Wang

**Affiliations:** ^1^Department of Intensive Care Unit, Guangzhou Twelfth People’s Hospital, Guangzhou, China; ^2^Key Laboratory of Occupational Environment and Health, Guangzhou Twelfth People’s Hospital, Guangzhou, China

**Keywords:** diquat, multi-organ dysfunction, rhabdomyolysis, shock, pulse indicator continuous cardiac output

## Abstract

The widespread use of diquat as a substitute for paraquat has led to an increase in poisoning deaths. A successful case of diquat poisoning complicated with rhabdomyolysis and shock was lacking. A 13-year-old previously healthy girl ingested 40 ml of diquat solution in a suicide attempt. The concentration of diquat in serum was 436.2 ug/L at 10 h after poisoning. The clinical course was characterized by progressive multi-organ dysfunction, particularly rhabdomyolysis and shock. The main treatments included intensive hemoperfusion combined with continuous renal replacement therapy (CRRT), drainage, and activated carbon adsorption. Meanwhile, accurate dilatation under the model of pulse indicator continuous cardiac output (PICCO) was essential for the successful treatment of shock. The serum concentration of diquat declined to 20 ug/L after 96 h of treatments. The patient was discharged from the hospital after 3 weeks of treatment without obvious symptoms. So far, this was the first successful case of diquat poisoning complicated with rhabdomyolysis and shock, which would enrich the experience of diquat poisoning treatment.

## Introduction

Pesticide poisoning is the leading cause of poisoning and accidental death in many developing countries. Diquat is a highly toxic bipyridine herbicide, and has led to remarkable increases in poisonings as a substitute for paraquat in recent years. The national poison data system of the United States revealed 2,128 cases of diquat poisoning between 1998 and 2013 (1). There was a clear correlation between the prognosis and intake, and the lethal dose for humans was 6–12 g (namely 56.10–112.20 ml diquat solution with 20% concentration), and there was no effective antidote (2). Acute diquat poisoning damaged the kidney, liver, and central nervous systems, and subsequent multiple-organ failure syndromes were the main cause of death (3). Diquat poisoning caused acute kidney injury (AKI) as much as 73.3%, which was higher than other types of pesticide poisoning (4–6). Rhabdomyolysis is rarely reported in diquat poisoning (7, 8), but it could largely aggravate kidney injury. Meanwhile, diquat poisoning with shock was extremely fatal (9), and no surviving cases had been reported so far (8, 10), indicating the need for more effective treatment options.

Hemodynamic monitoring could provide continuous and dynamic information about circulation, perfusion, and oxygenation in the tissues and organs, which could effectively prevent transfusion-associated circulatory overload (11). A previous study reported that invasive hemodynamic monitoring was used for the treatment of shock caused by organophosphorus poisoning (12). So far, the clinical experience of invasive hemodynamic monitoring in the treatment of shock caused by diquat poisoning is very lacking. Therefore, this work reported a severe case of diquat poisoning with rhabdomyolysis and shock, and highlighted the successful treatment using continuous renal replacement therapy (CRRT) and hemodynamic monitoring.

## Clinical course

**FIGURE 1:**
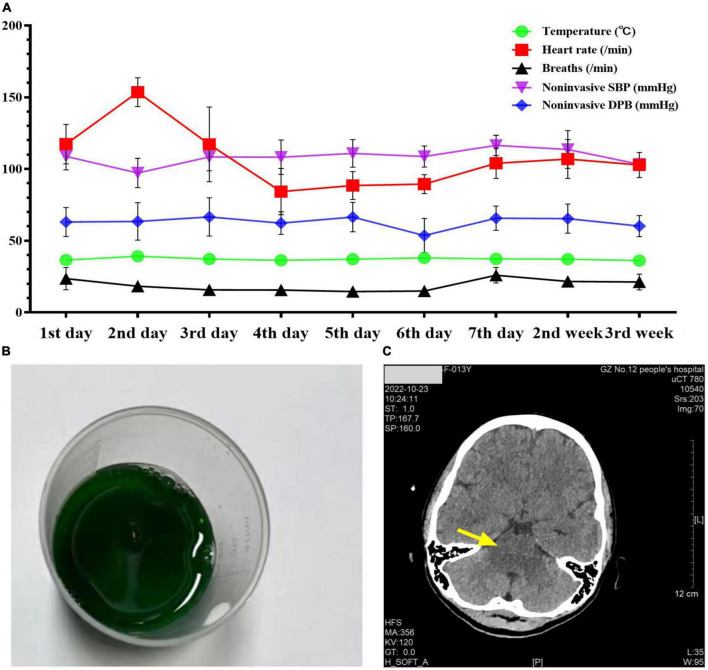
The patient’s vital signs, urine screening, and cranial CT examination during admission. Panel **(A)** was the changes in the patient’s vital signs during admission. Panel **(B)** was the positive urine fast screening of diquat (dark green). Panel **(C)** was the photograph of cranial CT examination on the 7th day after admission.

**FIGURE 2:**
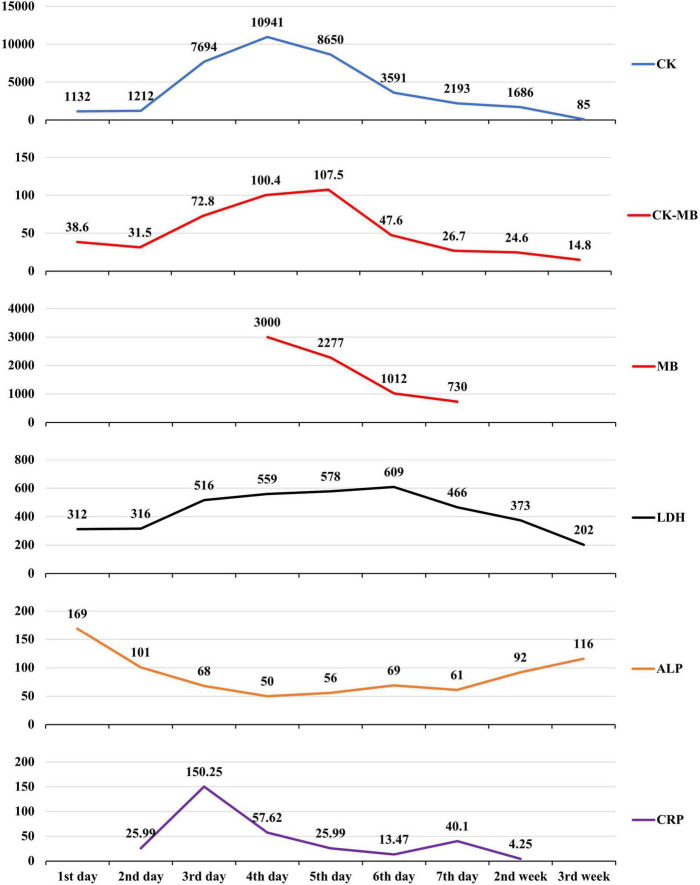
Changes in the patient’s main laboratory test results during admission.

A 13-year-old previously healthy girl ingested 40 ml of diquat solution in a suicide attempt. The patient developed paroxysmal pain in the upper abdomen and a sore throat, accompanied by vomiting yellow-green stomach contents several times. After 10 h, she was transferred to the intensive care unit of our hospital for further treatment. The vital signs and physical examination showed no significant abnormalities, except slight tenderness in the upper abdomen (Figure 1A). The serum concentration of diquat was 436.2 ug/L, and urine fast screening of diquat was positive (Figure 1B). Many blood tests were abnormal, especially creatinine (93 μmol/L), creatine kinase (1132 u/L), creatine kinase MB isoenzyme (38.6 u/L), and lactate dehydrogenase (312 u/L). Immediately, she accepted the intensive hemoperfusion combined with continuous renal replacement therapy (CRRT) in the model of continuous venovenous hemodiafiltration (CVVHDF), activated carbon adsorption and Sivelestat (Shanghai Huilun Life Science & Technology Co., Ltd., Shanghai, China) for anti-inflammation, and other supportive treatments. In the afternoon, the patient developed dyspnea, irritability, and confusion, suggesting toxic encephalopathy, which was supported by the subsequent screen of computed tomography (CT) (Figure 1C). The patient was given nasal tracheal intubation with ventilator-assisted ventilation in the model of PSIMV (FiO_2_ 45%, f 14 times/min, PC 16 cmH_2_O, PS 15 cmH_2_O, PEEP 3 cmH_2_O). On the 2nd day after admission, the patient developed an elevated body temperature, with a maximum body temperature of 40.1°C, accompanied by a heart rate of 145–179 beats/min. Blood pressure decreased progressively in the range of 55–68/28–35 mmHg, and urine volume also pronouncedly decreased. Procalcitonin 27.81 ng/ml. At the same time, hemodynamic detection using the model of pulse indicator continuous cardiac output (PICCO) showed CI 2.51 L/min/m^2^, SVRI 2465 dyn.s.m^2^/cm^5^, dPmax 1452 mmHg/s, GEDI 292 ml/m^2^, ITBI 364 ml/m^2^, ELWI 7 ml/kg, SVI 15 ml/m^2^, and GEF 23%. The above supported the diagnosis of hypovolemic shock, and anti-shock therapies, including dilatancy and fluid rehydration, were performed under PICCO monitoring immediately. Meanwhile, CRP 25.99 mg/L, IL-6 > 5000.0 ng/L, and PCT 11.72 ng/ml, indicating the diagnosis of systemic inflammatory response syndrome (SIRS). On the 3rd day after admission, the patient developed pain and swelling in both legs. The pronouncedly elevated levels of creatine kinase (the highest was 10941 u/L on the 4th day) and myoglobin (>3000.00 ug/L), which was diagnosed as rhabdomyolysis. After precise fluid rehydration and dilatation, the shock was corrected and urine volume recovered, and the myoenzyme spectrum decreased gradually (Table 1 and Figure 2). On the 5th day after admission, the serum concentration of diquat was cleared to less than 20 ug/L. On the 7th day after admission, the patient recovered well, including the spontaneous respiratory. Meanwhile, the indicators of auxiliary examination gradually stabilized. Three weeks after admission, the patient was discharged from the hospital without obvious discomfort.

**TABLE 1:** Changes in the patient’s laboratory test results during admission.

Index	Normal range	1st day	2nd day	3th day	4th day	5th day	6th day	7th day	2nd week	3rd week
WBC	5–12 × 10^9^	11.64	4.39	6.03	8.33	10.76	12.77	15.3	13.6	4.55
NEUT%	40–75%	89.7	74.7	67.8	74.9	82.6	80.4	72.5	77.9	53.9
HGB	105–145 g/L	114	123	124	78	80	69	65	65	87
PLT	140–440 × 10^9^/L	234	213	137	67	59	70	101	439	372
CRE	35–73 μmol/L	93	105	212	123	63	61	87	43	49
CRP	0–6 mg/L	NA	25.99	150.25	57.62	25.99	13.47	40.10	4.25	NA
CK	40–310 u/L	1132	1212	7694	10941	8650	3591	2193	1686	85
CK-MB	0–25 u/L	38.6	31.5	72.8	100.4	107.5	47.6	26.7	24.6	14.8
MB	28–72 ug/L	NA	NA	NA	>3000.00	2277	1012	730	NA	NA
LDH	120–250 u/L	312	316	516	559	578	609	466	373	202
AST	7–40 u/L	26.5	45.9	136.8	204.4	NA	NA	NA	52.9	19.3
ALT	7–40 u/L	10.5	20.7	44.7	71.9	116.1	126.8	98.7	70	29.5
ALP	125–700 u/L	169	101	68	50	56	69	61	92	116
IL6	0–7 ng/L	NA	>5000.00	67.65	7.83	6.05	71.87	26.35	NA	NA
PCT	0–0.046 ng/mL	0.089	11.72	27.81	16.39	10.76	3.84	1.44	NA	NA

WBC, white blood cell; NEU, neutrophil; HBG, hemoglobin; PLT, platelets; CRE, creatinine; CRP, C-reactive protein; CK, creatine kinase; CK-MB, creatine kinase-MB; MB, myoglobin; LDH, lactate dehydrogenase; AST, aspartate aminotransferase; ALT, alanine aminotransferase; ALP, alkaline phosphatase; IL6, interleukin 6; PCT, procalcitonin.

## Discussion

Our institution is the Guangzhou regional center of poison treatment, and had treated dozens of diquat and paraquat poisonings in recent years. So far, this was the first successful outcome of diquat poisoning complicated with rhabdomyolysis and shock. Although the poison dose was not very high, the ingested dose was lethal for her underweight (height 1.60 m, weight 41.5 kg, body mass index 16.21). Meanwhile, underweight patients easily developed unfavorable mortality trends due to shock, which was probably related to malnutrition (13). Poison removal is critical to clinical treatment, but the intensity of hemoperfusion was still controversial and lack of systematic verification (14, 15). A prospective study found that continuous venous hemofiltration (CVVH) combined with hemoperfusion could significantly improve the 90-day survival of paraquat poisoning (16). The worsening condition was observed in this patient after the routine hemoperfusion. In order to promote the removal of poisons in the early stage of poisoning, an intensive model of CVVHDF was adopted in the early stage to remove poisons as soon as possible, thus reducing subsequent organ damage. The symptoms improved significantly using the intensive hemoperfusion strategy, which was similar to a previous study (17). In the present case, low blood pressure, elevated blood lactic acid, poor tissue perfusion, and maintenance of blood pressure with high doses of vasoactive drugs supported the diagnosis of circulatory failure (18). Diquat rapidly distributed throughout the body, and largely damaged tissues and cells, and subsequent systemic inflammatory response, resulting in reduced effective circulating blood volume (19), which probably explained the cause of shock. According to the previous report on diquat poisoning combined with rhabdomyolysis, the shock was the main cause of death due to the damage to myocardial function (8). Especially, the high level of IL6 indicated the development of systemic inflammatory response syndrome (SIRS) (20). Decreasing blood pressure might be related to the increased permeability of capillary caused by SIRS, and the transfer of extracellular fluid to the tissue space after rhabdomyolysis (21). Fluid resuscitation under the guidance of PICCO could accurately evaluate blood volume, regulate hemodynamics, and blood gas analysis, which effectively relieved symptoms of ischemia and hypoxia (22). Therefore, this case supported that invasive hemodynamic monitoring should be implemented as early as possible to guide precise treatment for shock. Diquat causes strong toxic reactions such as lipid peroxidation, damaged the fluidity and permeability of the cell membrane structure, and ultimately leads to cell rupture and death (10), which explained the cause of rhabdomyolysis. Yin et al. reported that creatine kinase isoenzyme and myoglobin were the important risk factors of AKI caused by pesticide poisoning (23). In this case, the serum creatinine rose to 212 umol/L on the third day after admission, indicating that the patient had acute renal function damage. However, the acute renal injury was effectively mitigated by continuous hemofiltration dialysis, and the antishock treatment also prevented renal function damage in the early stage. Sivelestat was a new drug for acute respiratory distress syndrome (ARDS) that blocked the systemic inflammatory reaction by targeting the inhibition of neutrophil elastase (24). The toxicological mechanism of bipyridine herbicides, such as paraquat and diquat, has not been clarified yet, but the currently recognized mechanism is the oxidative stress pathway. Diquat could greatly stimulate mitochondrial tissues to produce a large number of oxygen-free radicals, and also activate neutrophils to release a large number of inflammatory mediators (25). Early use of anti-inflammatory agents was recommended according to the latest guidelines for sepsis management. The use of Sivelestat aimed to reduce the neutrophil release of inflammatory mediators, thereby reducing systemic inflammatory response (26). The outcomes of Sivelestat in the treatment of diquat poisoning were positive, which yet still needed more clinical evidences to confirm.

In conclusion, the clinical treatment and management of diquat poisoning were complex, particularly complicated with rhabdomyolysis and shock. The successful outcomes of this patient highlighted that intensive CRRT and PICCO were effective measures for poisoning and shock. Additionally, adolescent mental health is also worth attracting more attention and assistance.

## Data availability statement

The raw data supporting the conclusions of this article will be made available by the authors, without undue reservation.

## Ethics statement

The studies involving human participants were reviewed and approved by the Ethics Committee of Guangzhou Twelfth People’s Hospital. Written informed consent to participate in this study was provided by the participants’ legal guardian/next of kin. Written informed consent was obtained from the minor(s)’ legal guardian/next of kin for the publication of any potentially identifiable images or data included in this article.

## Author contributions

YC and ZO conceptualized and wrote the draft in consultation with ZW. RZ and ST collected and collated the data. RF and ZL analyzed and visualized the data. All authors reviewed the manuscript and approved the submitted version.
